# Flow based single cell analysis of the immune landscape distinguishes Barrett’s esophagus from adjacent normal tissue

**DOI:** 10.18632/oncotarget.26911

**Published:** 2019-06-04

**Authors:** Moen Sen, Friedrich Hahn, Taylor A. Black, Maureen DeMarshall, Warren Porter, Eileen Snowden, Stephanie S. Yee, Frances Tong, Mitchell Ferguson, Emylee N. Fleshman, Hiroshi Nakagawa, Gary W. Falk, Gregory G. Ginsberg, Michael L. Kochman, Rainer Blaesius, Anil K. Rustgi, Erica L. Carpenter

**Affiliations:** ^1^ Division of Hematology and Oncology, Department of Medicine, Abramson Cancer Center, Perelman School of Medicine, University of Pennsylvania, Philadelphia, Pennsylvania, USA; ^2^ Department of Genomic Sciences, BD Technologies and Innovation, Research Triangle Park, Durham, North Carolina, USA; ^3^ Division of Gastroenterology, Department of Medicine, Abramson Cancer Center, Perelman School of Medicine at the University of Pennsylvania, Philadelphia, Pennsylvania, USA

**Keywords:** Barrett’s esophagus, microenvironment, immune cells, single cell, flow cytometry

## Abstract

Barrett’s esophagus (BE) is metaplasia of the squamous epithelium to a specialized columnar epithelium. BE progresses through low- and high-grade dysplasia before developing into esophageal adenocarcinoma. The BE microenvironment is not well defined. We compare 12 human clinical BE and adjacent normal squamous epithelium biopsies using single cell immunophenotyping by flow cytometry. A cassette of 19 epithelial and immune cell markers was used to detect differences between cellular compartments in normal and BE tissues. We found that the BE microenvironment has an immunological landscape distinct from adjacent normal epithelium. BE has an increased percentage of epithelial cells with a concomitant decrease in the percentage of immune cells, accompanied by a shift in the immune landscape from a predominantly T cell rich microenvironment in normal tissue to a B cell rich landscape in BE tissue. Hierarchical clustering separates BE and normal samples into two discrete groups based upon our 19-marker panel, but also reveals unexpected, shared phenotypes for three patients. Our results suggest that flow based single cell analysis may have the potential for revealing clinically relevant differences between BE and normal adjacent tissue, and that surface immunophenotypes could identify specific subpopulations from dysplastic tissue for further investigation.

## INTRODUCTION

The esophageal mucosa is a prototype stratified squamous epithelium, comprising proliferative basal cells that reside upon the basement membrane and migrate towards the luminal surface undergoing a differentiation program from suprabasal cells to superficial squamous cells [1]. Eventually, the superficial layer of cells desquamates and the gradient of proliferative-differentiated cells renews on a regular basis. Such a gradient is regulated exquisitely through a network of transcriptional factors, but is subject to noxious stimuli, such as acid and bile reflux, infectious microorganisms, and malignant transformation [2].

Acid and bile reflux induce epithelial cell oxidative stress and DNA damage, and when combined with inflammation in the subepithelium or submucosa, induce an adaptive change of the normal stratified squamous epithelium to intestinal epithelial metaplasia. This metaplastic process is referred to as Barrett’s esophagus or Barrett’s metaplasia [1]. While metaplasia may remain dormant, a subset of metaplastic cells can undergo a transition to low-grade dysplasia and high-grade dysplasia with culmination in esophageal adenocarcinoma. Barrett’s esophagus is estimated to affect 6-8 million individuals in the United States. It is estimated that the annual risk of progression from Barrett’s esophagus to esophageal adenocarcinoma is approximately 0.33% per year [3]. If one incorporates both high-grade dysplasia and esophageal adenocarcinoma as an endpoint of progression from Barrett’s esophagus, it is believed the incidence is about 0.9–1.0% per year [4, 5].

Established risk factors for the emergence of Barrett’s esophagus include increasing age, white Caucasian males, central obesity, and acid and bile reflux. The preponderance of Barrett’s esophagus is sporadic in nature, however, a small proportion may have a hereditary basis. Mechanistically, epithelial cell intrinsic factors have been identified that underlie the pathogenesis of Barrett’s esophagus, and involve changes in the genomic landscape. A key driver is TP53 mutation [6–8], apart from epigenetic alterations and copy number variation. Epithelial cell extrinsic factors involve the induction of various cytokines, chemokines and growth factors. Inflammation has been implicated in the pathogenesis of a number of cancers and studies have established the role of an aberrant microenvironment in aberrant growth and transformation. That being said, the precise composition of the immune landscape in Barrett’s esophagus is not known, thereby forming the rationale of this human-based study and analysis. A comprehensive characterization of the BE immune microenvironment would thus permit a new perspective on preventive and therapeutic strategies in Barrett’s esophagus and its progression to adenocarcinoma. In addition, many challenges remain predicting which patients will progress to dysplasia and then adenocarcinoma. An understanding of immune and epithelial cells will aid in the potential implementation of preventive, early detection and therapeutic strategies, all synergized to disrupt the continuum from dysplastic BE to adenocarcinoma.

## RESULTS

### Patient characteristics and study design

**Figure 1:**
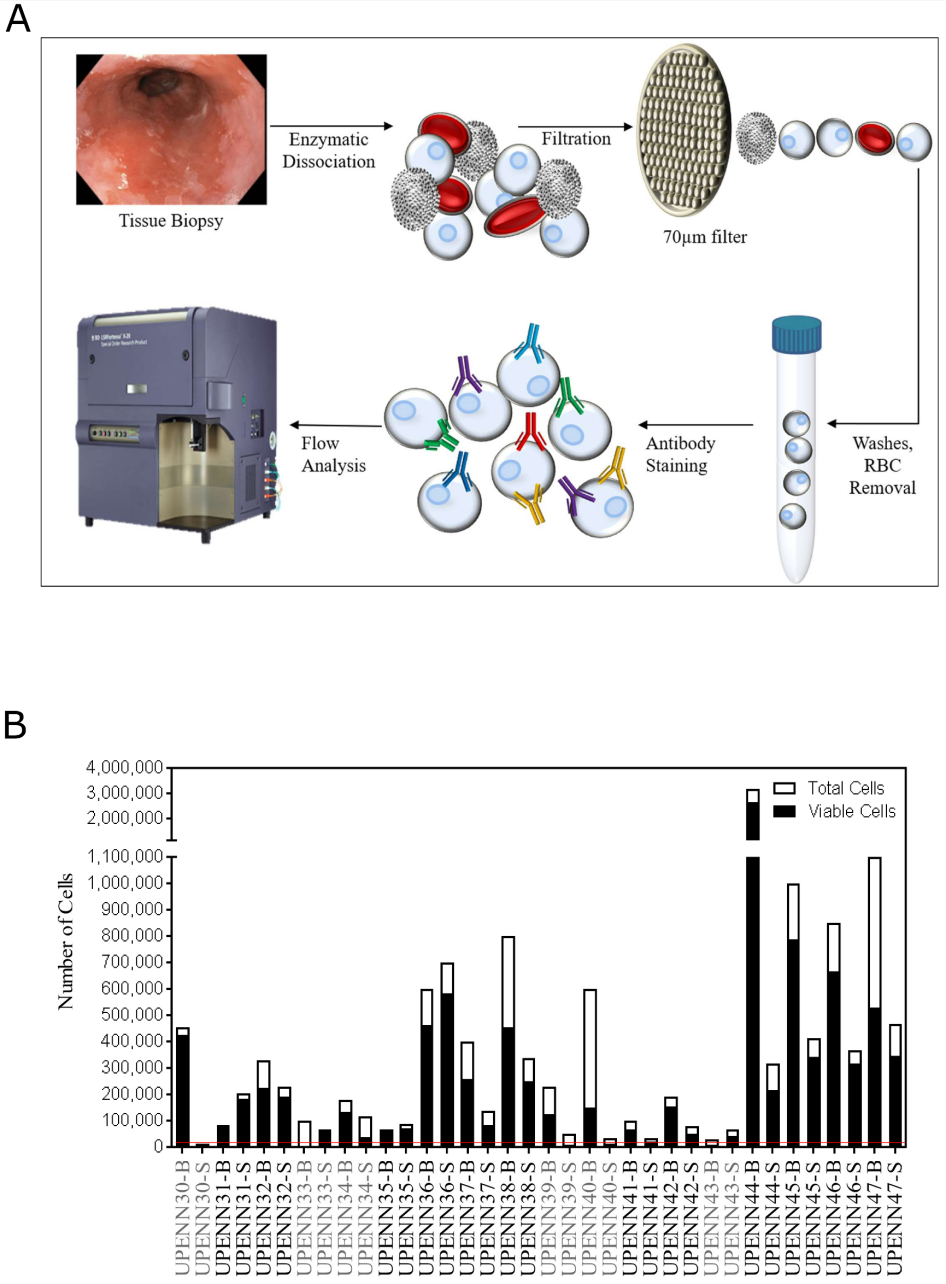
Schematic overview of single cell analysis for tissue immunophenotyping. **(A)** Matched diseased and adjacent normal tissue biopsies are dissociated into single cell suspensions by enzymatic dissociation. Cells are passed through a 70μm filter and RBCs are lysed and washed away. Cell numbers and viability are estimated using Vi-CELL XR automated cell viability analyzer. Cells are then labeled with fluorochrome-conjugated antibodies so that immunophenotypic signatures can be analyzed by flow cytometry. **(B)** Total and viable cell yields after tissue dissociation. Threshold for inclusion into the analysis was set at 20,000 total viable cells (red line), with 5 samples (UPENN30, UPENN33, UPENN39, UPENN40 and UPENN 43) excluded for insufficient viable cells. UPENN34 was not able to be stained with all markers so was also excluded. Excluded samples are highlighted in grey.

Biopsies of Barrett’s esophagus and adjacent normal squamous epithelium were dissociated enzymatically (range of 10.1 mg – 40.0 mg; median of 23.3 mg), passed through a 70μm filter, and treated with RBC lysis buffer to obtain a single cell suspension (Figure 1A), which was then probed with a Live/Dead cell stain to assess viability. The number of viable cells ranged from 0 - 2,643,200 cells (median of 154,672 cells) for the 18 pairs of samples. The total number of cells ranged from 15,000 - 3,200,000 (median of 217,500 cells), thus the percentage of viable cells ranged from 0.0 - 94.3% (median = 72.6%). Five patients had fewer than 20,000 viable cells for one or both of their matched samples and were therefore excluded from further analysis. One sample pair that could not be stained with all required markers was also excluded from further analysis (Figure 1B). Of the 12 samples used for further analysis, the majority (66.7%) were male and all (100%) were white (Table 1). The median age of the cohort was 61 years (range 53 – 81) and median BMI was 30.6 (23.5 – 35.0). The rate of active cigarette use in the cohort was 8.3%, 25% were former smokers, and 66.7% were never smokers. Eight patients (66.7%) had intestinal metaplasia only without dysplasia, one (8.3%) patient had low-grade dysplasia, one (8.3%) patient had high-grade dysplasia, two patients (16.7%) had adenocarcinoma, and all patients had a documented Prague classification (Supplementary Table 1).

**Table 1:** Patient characteristics.

	n (%)
	All patients (n = 12)
Median age	
Median	61 (53 – 81)
Sex	
Male	8 (67)
Female	4 (33)
Race	
White	12 (100)
Other	0 (0)
Months from Diagnosis	
Median	33.8 (2.0 – 84.3)
BMI	
Median	30.6 (23.5 – 35.0)
Smoking status	
Active	1 (8)
Former	3 (25)
Never	8 (67)
Pathology	
Intestinal Metaplasia	8 (67)
Low Grade Dysplasia	1 (8)
High Grade Dysplasia	1 (8)
Adenocarcinoma	2 (17)

### Differential biomarker expression in Barrett’s esophagus and adjacent normal tissues

**Figure 2:**
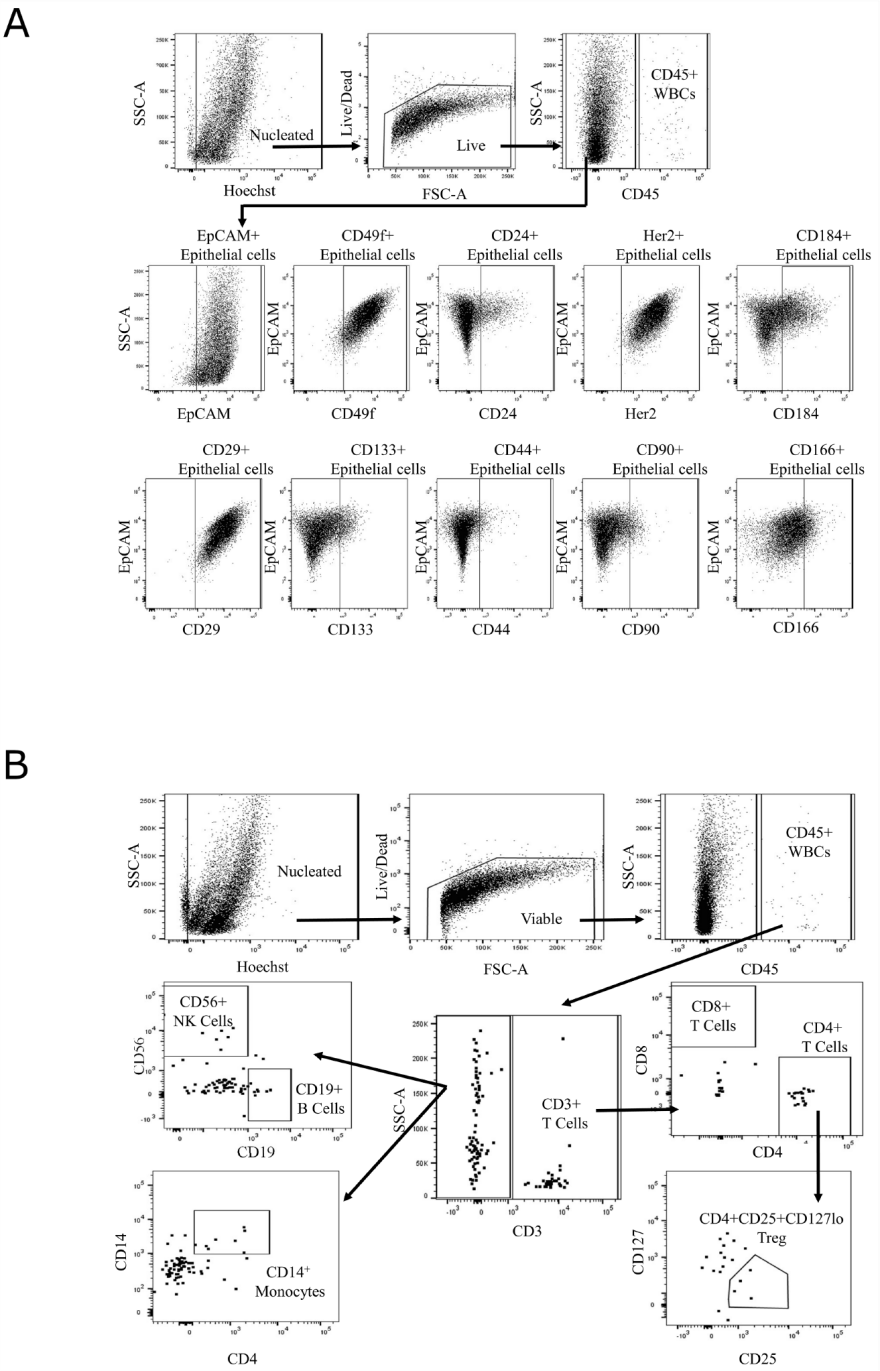
Gating strategy for single-cell immunophenotyping. Gating strategy for epithelial **(A)** and immune cell **(B)** marker panel on LSRFortessa X-20 with representative image of Barrett’s esophagus tissue (UPENN42-B) cell suspension. Hoechst and amine staining are used to eliminate cellular debris and non-viable cells present in the sample. CD45-negative cells are analyzed for a set of epithelial cell markers. CD45-positive cells are analyzed for a set of immune cell markers.

**Figure 3:**
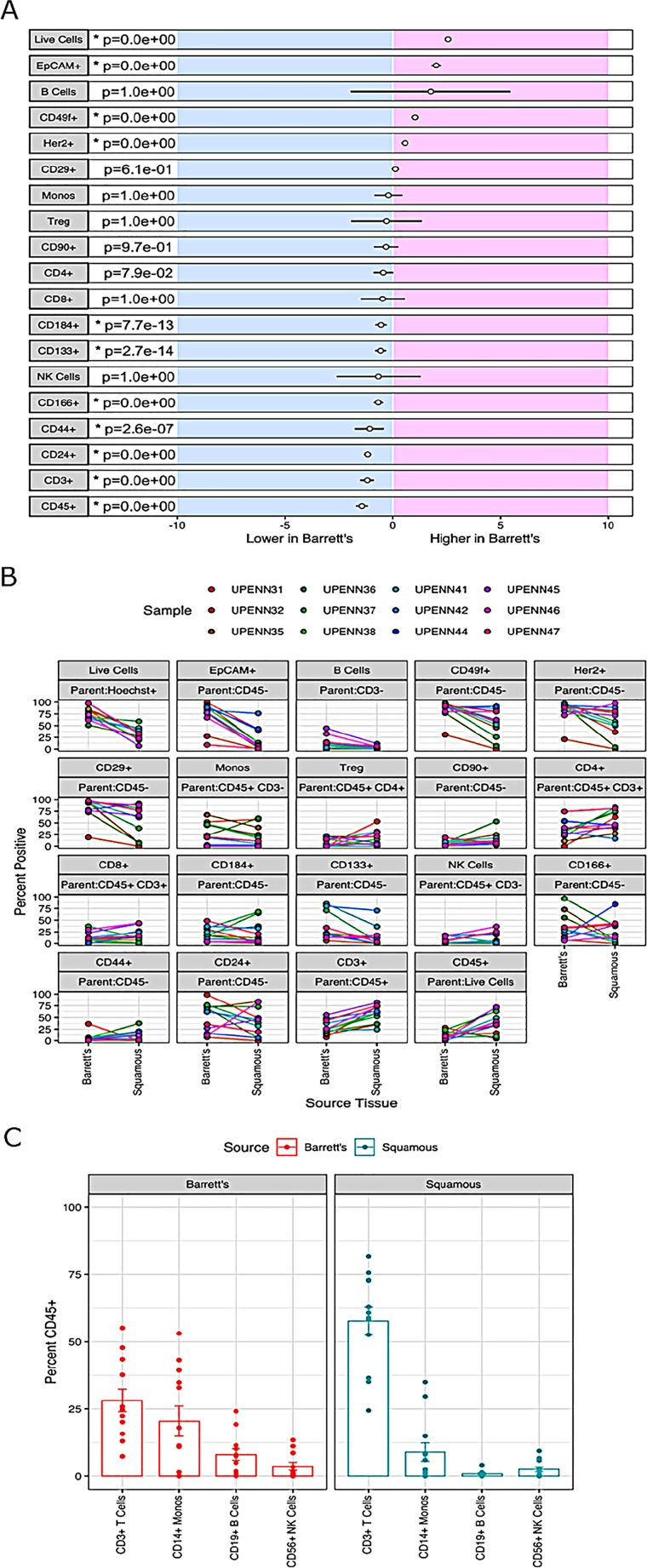
Differential biomarker expression in Barrett’s esophagus and adjacent normal tissues. **(A)** Results for a generalized linear model (GLM) used to generate estimates for each tissue-surface marker interaction. For each marker, the mean difference between BE and normal squamous is indicated by the circle and the 95% confidence interval of the differences by the black line. The general linear hypothesis testing approach (GLHT) was used to correct for simultaneous testing. Markers whose confidence intervals do not overlap with zero are significantly different between Barrett’s and Squamous tissue types (p-values listed and significantly different markers denoted by asterisks). **(B)** Percent positive of the parent population for each surface marker in each individual normal/BE pair. For each marker, the parent population is listed in the top grey row and the marker of interest in the bottom grey row above each plot. **(C)** Comparison of immune marker profiles of BE and normal Squamous tissue. Immune subsets of CD45+ WBCs were determined based on the gating scheme shown in Figure 2B, and including markers for: CD3+ T cells, CD3-CD19+ B cells, CD3-CD14+ monocytes, and CD3-CD19-CD56+ NK cells. The height of each bar represents the median percent of CD45+ cells in each immune subset.

For comprehensive immunophenotyping, we compiled a cassette of canonical epithelial targets (EpCAM, CD24, CD44, CD49f, Her2/neu (Her2), CD133, CD90, CD166, CD184, and CD29) and immune cell markers (CD3, CD45, CD127, CD56, CD4, CD8, CD14, CD25, and CD19) (Supplemtary Table 2) to discern differences across normal and BE tissues (gating strategy shown in Figure 2). We (abstract 2113, AACR 2018) and others have previously shown these markers to be unaffected by tissue processing [9, 10]. To test if the differences in cellular composition of BE and normal adjacent squamous epithelium tissue were significant, a generalized linear model (GLM) was used to generate estimates for each tissue/surface marker interaction. This was followed by correcting for multiple testing, using the general linear hypothesis testing approach (GLHT), which generated the mean and confidence intervals for the difference between BE and normal squamous estimates for each surface marker (Figure 3A). Parallel coordinate plots for each marker are shown in Figure 3B, in descending order as ranked by the GLM for expression in BE tissue. BE tissue had a significantly higher percentage of live cells as compared to normal squamous tissue. Among the epithelial markers measured, BE samples are characterized by a higher percentage of epithelial cells (CD45-, EpCAM+) including cells expressing Her2, a marker of BE and esophageal adenocarcinoma, and the cell adhesion marker CD49f. Expression of CD133, CD184, CD166, CD24, and CD44 were all significantly lower in BE samples as compared to normal tissue, while there were no significant differences in CD29 or CD90.

Next, we investigated the composition of the immune microenvironment of BE as compared to matched adjacent normal squamous epithelium using markers against T cells (CD3, CD4, and CD8), B cells (CD19), monocytes (CD14), NK cells (CD56), and T-regulatory cells (Tregs; CD25 and CD127). While BE tissue had an increased percentage of B cells as compared to adjacent normal tissue, this difference did not reach statistical significance due to the wide range of expression among BE samples. There was also no significant difference in the percentage of monocytes, Tregs, CD4+ T cells, CD8+ T cells, or NK cells between BE and adjacent normal squamous tissue (Figure 2). In comparison with BE, adjacent normal squamous tissue had an increased percentage of CD45+ immune cells and CD3+ T cells. CD3+ T cells, followed by monocytes, comprised the two largest subsets of CD45+ cells detected in both BE and normal tissue (Figure 3C). Taken together, these results suggest that significant differences exist in marker expression at a single cell level between matched diseased and adjacent normal biopsies from Barrett’s esophagus patients.

### Marker panel facilitates accurate clustering of BE from normal tissue

**Figure 4:**
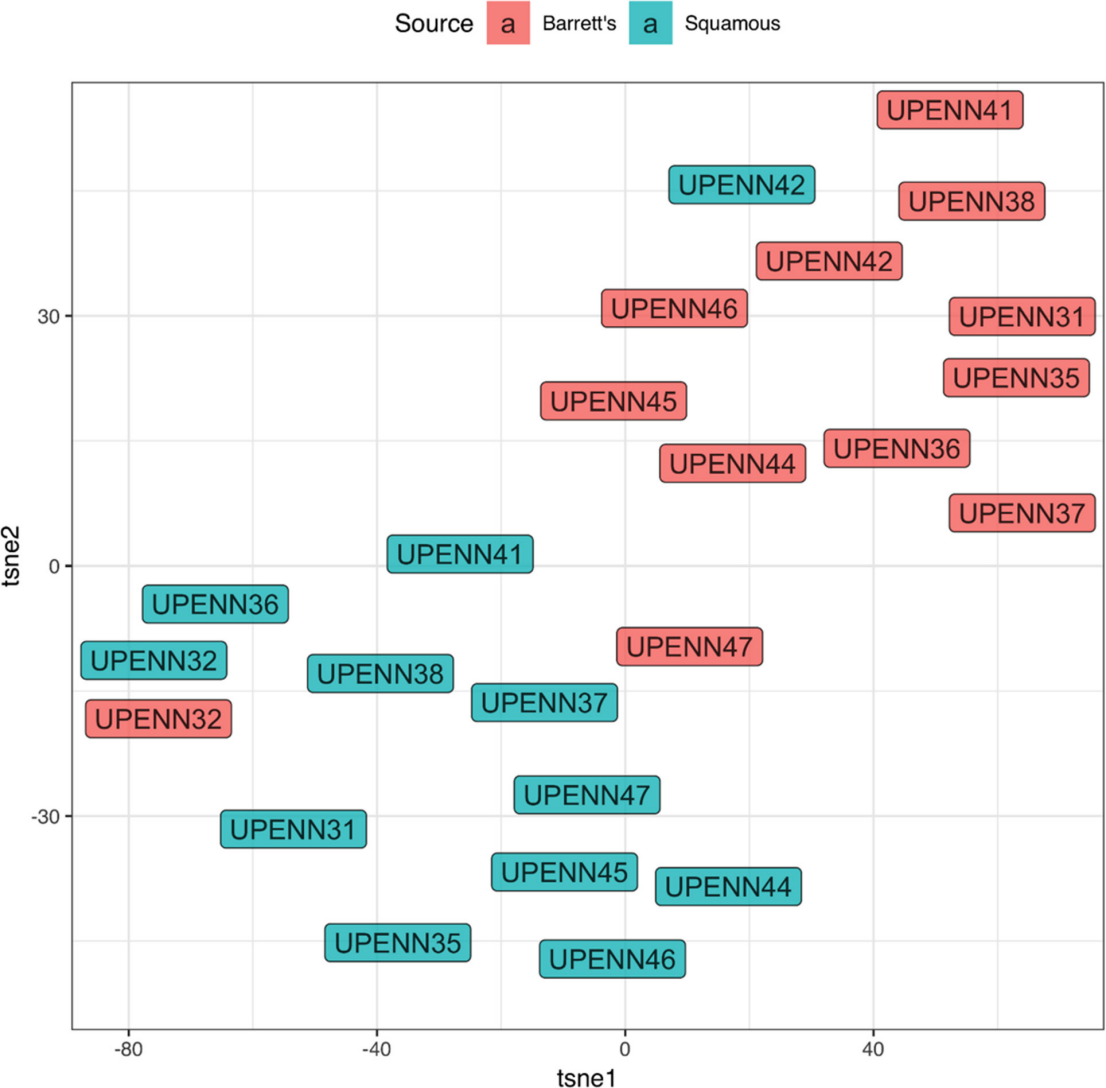
t-distributed stochastic neighbor embedding (t-SNE) of marker expression of Barrett’s esophagus vs. normal adjacent squamous tissues. Phenotypic clustering of BE and squamous tissues is displayed as a t-SNE plot.

**Figure 5:**
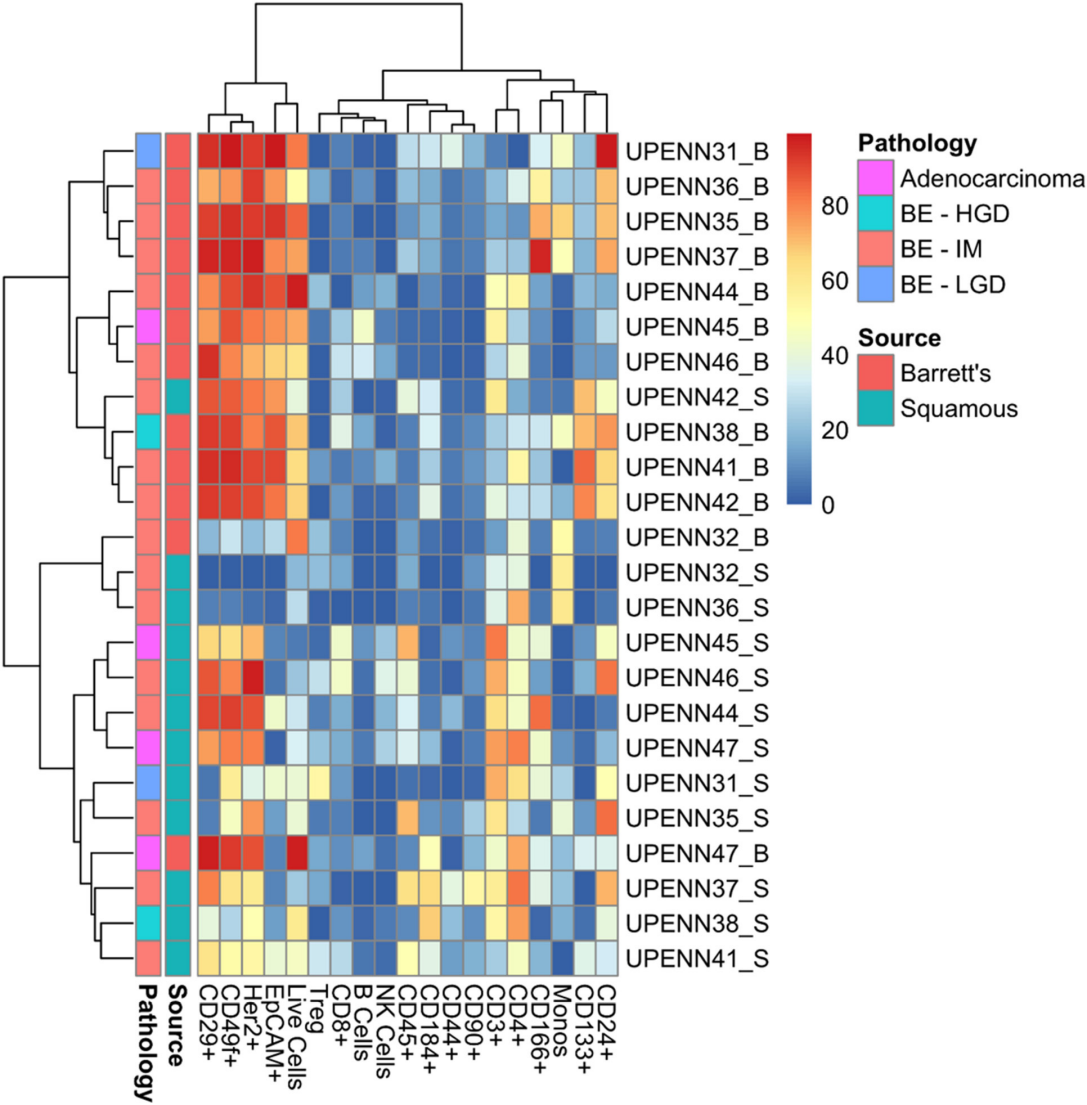
Heatmap with distance trees displaying clusters of individual samples using surface markers or cell types. Unsupervised hierarchical clustering (complete clustering method using Euclidean distance) of marker expression in cells from BE and normal squamous tissue as detected by flow cytometry. Scale bar refers to percent positive for each sample/marker combination. BE, Barrett’s esophagus; HGD, High Grade Dysplasia; IM, Intestinal Metaplasia; LGD, Low Grade Dysplasia.

To assess whether our marker panel could accurately distinguish between BE and matched normal tissue, we next used t-distributed stochastic neighbor embedding (t-SNE) as shown in Figure 4. t-SNE showed that BE and normal squamous tissue formed separate clusters but also revealed unexpected similar phenotypes. The BE tissues of patient UPENN32 and UPENN47 (patient with confirmed adenocarcinoma), clustered with normal squamous tissues, and the normal squamous tissue of patient UPENN42 clustered with BE tissues. As shown in Figure 5, hierarchical clustering supports this grouping. A heatmap with distance trees was able to recapitulate the clustering of BE samples away from normal squamous samples into two discrete groups (Figure 5), and as seen with t-SNE analysis, normal adjacent squamous tissue from patient UPENN42 clustered with BE tissues while BE tissues from patient UPENN47 and UPENN32 clustered with normal squamous tissues. Hierarchical clustering revealed low expression of EpCAM in BE tissue for UPENN47 and UPENN32, consistent with the EpCAM levels of other patients’ squamous tissue, and high expression of EpCAM in the normal squamous tissue for UPENN42, consistent with EpCAM levels for other patients’ BE tissue. This clustering analysis suggests that the selected panel of 19 markers can effectively distinguish BE and normal tissue for the majority of patients in our cohort.

### Selection of markers most important for distinguishing phenotypic states

**Figure 6:**
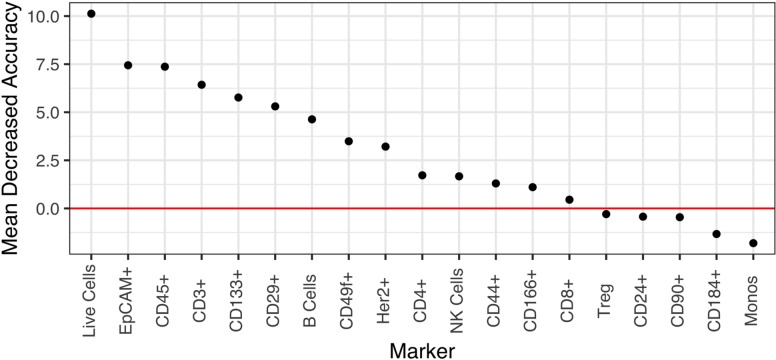
Marker importance as measured by the mean decrease in accuracy for each biomarker in a random forest model. The model directly measures the impact of each feature on accuracy of the model. Markers whose values are greater than zero, i.e., above the red horizontal line, positively contribute to the model accuracy.

We reasoned that a 19-marker flow panel might prove unwieldy for eventual deployment in a clinical flow cytometry lab where 5- or 10-marker panels are more routinely used. To determine whether the accuracy of a smaller panel would be sufficient and to identify those markers that are essential for accurately distinguishing BE from normal adjacent tissue, we next employed the random forest (RF) model to rank the 19 markers according to their predictive power of a biological phenotype. As shown in Figure 6, 14 of the 19 markers have a positive Mean Decreased Accuracy (MDA) score and positively contribute to the model’s accuracy. To determine the accuracy of our marker panel as well as subsets of those markers, we next used recursive feature elimination (RFE) in an iterative fashion, removing the lowest performing predictors after each iteration. Performing RFE for panels ranging from 5 to 19 markers, we found that accuracy varied only slightly from a low of 0.8167 for 12 markers to a high of 0.8778 for 13 markers. The accuracy of a 5, 8 or 9 marker panel was very similar to the accuracy of the 13 marker panel at 0.8611. These results suggest that a smaller, more feasibly sized panel of 5 markers would achieve similar accuracy as the full 19-marker panel (accuracy 0.8444). The minimal differences between the accuracy of the marker sets is most probably because the top markers are shared in every combination of markers used in the RFE and are likely driving the accuracy in each of the marker sets (Supplementary Table 3).

## DISCUSSION

Barrett’s esophagus represents metaplasia of the esophagus that can progress through stages of low- and high-grade degrees of dysplasia to esophageal adenocarcinoma. Previous studies have strongly implied a role for inflammation in the pathogenesis of BE [11–14]. Fitzgerald *et al.* analyzed bulk cell mRNA for a limited panel of cytokines and reported the presence of an inflammatory cytokine gradient in BE with higher expression of pro-inflammatory cytokines IL-8 and IL-1β at the new squamocolumnar junction [15]. Another group studying the immune cell composition of BE compared the Th1 and Th2 effector cell infiltrate of patients with BE to that of patients with reflux esophagitis (RE). Immunohistochemical analysis revealed that the proportion of Th2 effector cells (plasma cells and mast cells) was higher in BE than RE. By contrast, the immune composition of RE had a proinflammatory Th1 (CD8+ T cell) signature [14]. However, this study did not comprehensively analyze other immune cell subsets. A comprehensive and precise analysis of the composition of the immune and epithelial cell landscape in Barrett’s esophagus compared to normal squamous tissue has been lacking, thus providing the rationale for our studies. Utilizing single-cell flow analysis and probing for markers of T cells, B cells, monocytes, NK cells, and Treg cells, as well as epithelial markers, our study identifies marked differences in the surface immunophenotypes of matched BE and normal adjacent tissue. To our knowledge, our study is the first deep immunophenotypic characterization of the microenvironment of both BE and normal adjacent squamous tissue.

Inflammation, and more specifically shifts from a chronic cell-mediated environment to a humoral phenotype, has been thought to facilitate malignant growth by repression of cellular immunity and surveillance[16–21]. We report a decrease in the T cell population from normal squamous tissue to BE tissue that is accompanied by an increase in B cells, as the above studies would predict. Shifts from a T cell-rich microenvironment to a more B cell dominated landscape may potentially predict disease progression, although larger studies would have to be done to confirm this. In addition, these results highlight important unanswered questions about the molecular pathogenesis of BE. We demonstrate that an increase in B cells is accompanied by an increase in CD49f+ and Her2+ epithelial cells, suggesting that crosstalk between the epithelial and immune cell compartment may be an important factor in the pathogenesis of BE. Indeed, work by Derks *et al*. characterizes expression of the PD-1 ligand PD-L2 expression in epithelial BE and adenocarcinoma cells. Further *in vitro* work in adenocarcinoma cell lines demonstrates that Th2 cytokines, characteristically observed in BE, can augment PD-L2 expression [22]. Thus, our study paves the way for identification of shifts in cellular composition across the spectrum of disease, leading to novel hypotheses regarding disease pathogenesis. This work also demonstrates the feasibility of deep immunophenotyping to investigate the molecular crosstalk between specific epithelial and immune cell compartments and the role of epithelial cells in mediating the inflammatory response in BE. While expanded studies are required, it is tempting to speculate that strategies to disrupt the infiltration of B cells and/or augment the persistence of effector T cells may identify new therapeutic strategies to ameliorate progression of BE to dysplastic states and beyond to adenocarcinoma.

Our study also examines the expression patterns of epithelial cell markers. Previous immunohistochemical studies by Ecker *et al*. demonstrated that high-grade dysplasia in BE overexpress the HER family of receptor tyrosine kinases as compared to low-grade dysplastic lesions [11]. Additionally, Her2 expression in high grade dysplasia correlated with degree of dysplasia and disease progression [13, 23, 24]. Our results expand on these previous findings and demonstrate that a higher percentage of BE cells express Her2 across various stages of metaplasia, implicating a role for Her2 in the pathogenesis of BE and its progression to adenocarcinoma. When these epithelial markers were combined with immune cell markers for a panel of 19 total markers, this yielded a signature that could accurately distinguish normal from diseased tissue. While flow panels of this size or larger can be easily analyzed on a research basis, this number of markers might be unwieldy for adaptation to a clinical testing environment. Further analysis identified a 5 marker panel with similar accuracy for distinguishing normal from adjacent diseased tissue that could be readily deployed on 3- or 4-laser flow cytometry instruments. While this demonstrates the flexibility of our approach, additional experiments with larger numbers of patients will need to be done to determine the optimal combination of markers. Taken together, our results demonstrate the feasibility of deep phenotyping for comprehensive disease characterization and the eventual development of clinically relevant diagnostic tools and therapeutic strategies.

## MATERIALS AND METHODS

### Patient cohorts

Our cohort consisted of eighteen Barrett’s esophagus patients. Biopsies were obtained from Barrett’s esophagus, as defined by endoscopic appearance (confirmed by histopathology) and adjacent normal squamous epithelium. Viable cells were obtained from dissociated tissues (Figure 1A) and samples with fewer than 20,000 viable cells were excluded from further analysis. Deep flow cytometric phenotypic analysis requires multiple levels of sub-gating, thus we selected a cut-off of 20,000 viable cells. Five samples (UPENN30, UPENN33, UPENN39, UPENN40 and UPENN 43) were excluded for having a viable cell yield below this threshold (Figure 1B). Sample UPENN34 was excluded for technical problems with surface marker staining. The remaining 12 patients with matched biopsies from BE and adjacent normal squamous epithelium were included in our study (Table 1). This study was approved by the University of Pennsylvania Institutional Review Board (IRB).

### Esophageal tissue extraction and dissociation

Biopsies from Barrett’s esophagus and matched normal adjacent squamous tissue were collected endoscopically and immediately stored in sterile DPBS (Corning, Corning, NY, USA) on ice. Each matched tissue was treated identically such that the normal tissue could serve as an internal control for the Barrett’s esophagus tissue. De-identified tissue specimens were received by research staff within one hour of collection. Specimens were immediately weighed, placed in HypoThermosol FRS Preservation Solution (Sigma-Aldrich Chemical Company, St Louis, MO, USA), and shipped overnight on ice to BD Technologies and Innovation. Tissue was dissociated enzymatically into single cells as described previously [25–28]. Briefly, BE and adjacent normal biopsies were decanted through a 70μm filter and the supernatant was stored (workflow shown in Figure 1A). Biopsies were then transferred by forceps to a vessel with reconstituted BD Horizon™ Dri Tumor & Tissue Dissociation Reagent (BD Biosciences, San Jose, CA) and placed in a 37^o^C water bath for 30 minutes with frequent agitation. The enzymatic reaction was stopped by rinsing in PBS (Cellgro, Manassas, VA) with 1% Bovine Serum Albumin and EDTA (Sigma-Aldrich Chemical Company, St. Louis, MO), and the cell suspension pooled with the stored supernatant was filtered again through a 70 μm filter. After centrifugation, if blood was visible in the pellet, the samples were treated with ACK Buffer (Life Technologies, Grand Island, NY) to remove contaminating red blood cells, rinsed with PBS, centrifuged, and then re-suspended in Pre-Sort Buffer (BD Biosciences, San Jose, CA). Cell number and viability were estimated using Vi-CELL XR automated cell viability analyzer (Beckman Coulter, Indianapolis, IN).

### Cell staining for flow cytometry and analysis

Following dissociation, single-cell suspensions were incubated for 30 minutes on ice in FcR Blocking Reagent (Miltenyi Biotec, Auburn, CA), then diluted in PBS and evenly distributed into wells of a 96-well plate containing viability dye (LIVE/DEAD Fixable Aqua Dead Cell Stain reagent, Invitrogen, Eugene, OR) and Hoechst 33342 (Invitrogen, Eugene, OR) at 0.2μg/mL for viable nucleated cell identification. Fluorochrome-conjugated monoclonal antibodies were added and cells were incubated for 30 minutes at room temperature in the dark, then washed twice in PBS before acquisition on a BD flow cytometry cell analyzer (detailed list of staining antibodies in Supplementary Table 2). The first five pairs of samples (UPENN31, UPENN32, UPENN 35, UPENN36 and UPENN37 ) were run on a BD LSRII using a 10-color panel; the remaining 7 pairs of samples (UPENN38, UPENN41, UPENN42, UPENN44- UPENN47) were run on a BD LSRFortessa X-20 analyzer. The transition to the X-20 instrument allowed us to include an additional marker, CD14 FITC, and to increase the number of markers per well, thus reducing the number of wells needed for testing. For each sample, the total viable cells were divided evenly between the wells to be analyzed. Due to sparse cellularity we were unable to run fluorescence minus one (FMO) controls, and we used matched normal tissue populations and normal donor RBC-lysed peripheral whole blood samples stained in parallel with the test samples to act as experimental controls and as a reference for setting gates. Analysis was performed using FACSDiva software (version 6.1.3 LSRII or version 8.1 LSRFortessa X-20). Secondary analysis and graphical representations of the data were performed using R [R Core Team (2017). R: A language and environment for statistical computing. R Foundation for Statistical Computing, Vienna, Austria. URL https://www.R-project.org/]. For data modeling we used marker combinations for established, functionally distinct cell types (T cells, B cells, monocytes, NK cells, and T-regulatory T cells) and positive % of live cells for all other individual markers.

### Statistical analysis

To determine which markers are important in distinguishing BE vs. squamous tissues, we used two different approaches. First, to test if the differences in cellular composition of BE and normal adjacent squamous epithelium were significant, a generalized linear model (GLM) with a logit link was used to generate estimates for each tissue/surface marker interaction (Figure 3A). This was followed by correcting for multiple testing, using the general linear hypothesis testing approach (GLHT), which generated the mean and confidence intervals for the difference between BE and normal squamous estimates for each surface marker. Second, a random forest model was used to generate a measure of importance of each marker to correctly classify the sample types as either BE or squamous. Previous studies have utilized the RF model to rank genes according to their predictive power for leukemia, prostate and colon cancer [29]. Markers that have higher values are more important to the model for accurately predicting tissue disease state. Recursive feature elimination (RFE) is a feature selection method that fits a model and iteratively removes the weakest feature (or features) until the specified number of features is reached. We tested the accuracy of our RF model using the top 5 to 14 markers from our original 19 marker RF model. t-Distributed Stochastic Neighbor Embedding (t-SNE) is a non-linear dimensionality reduction algorithm used for exploring high dimensional data in two (or three) dimensional space, suitable for human observation.

## SUPPLEMENTARY MATERIALS FIGURE AND TABLE



## Abbreviations

BE: Barrett's esophagus
BMI: Body mass index
FMO: Fluorescence minus one
GLHT: General linear hypothesis testing approach
GLM: Generalized linear model
Her2: Her2/neu
HGD: High Grade Dysplasia
IM: Intestinal Metaplasia
IRB: Institutional review board
LGD: Low Grade Dysplasia
MDA: Mean Decreased Accuracy
Monos: Monocytes
NE: Normal esophagus
RBC: Red blood cell
RE: Reflux esophagitis
RF: Random Forest
RFE: Recursive Feature Elimination
t-SNE: t-distributed stochastic neighbor embedding
WBC: White blood cell

## References

[1] Giroux V, Rustgi AK. Metaplasia: tissue injury adaptation and a precursor to the dysplasia–cancer sequence. Nat Rev Cancer. 2017; 17: 594-604. 10.1038/nrc.2017.68. . 28860646 PMC5998678

[2] Morrisey EE, Rustgi AK. The Lung and Esophagus: Developmental and Regenerative Overlap. Trends Cell Biol. 2018; 28: 738–48. 10.1016/j.tcb.2018.04.007. . 29871822 PMC6102049

[3] Desai TK, Krishnan K, Samala N, Singh J, Cluley J, Perla S, Howden CW. The incidence of oesophageal adenocarcinoma in non-dysplastic Barrett’s oesophagus: a meta-analysis. Gut. 2012; 61: 970-976. https://gut.bmj.com/content/61/7/970.abstract 10.1136/gutjnl-2011-300730. . 21997553

[4] Sikkema M, de Jonge PJ, Steyerberg EW, Kuipers EJ. Risk of Esophageal Adenocarcinoma and Mortality in Patients With Barrett’s Esophagus: A Systematic Review and Meta-analysis. Clin Gastroenterol Hepatol. 2010; 8: 235–44. 10.1016/J.CGH.2009.10.010. . 19850156

[5] Yousef F, Cardwell C, Cantwell MM, Galway K, Johnston BT, Murray L. The Incidence of Esophageal Cancer and High-Grade Dysplasia in Barrett’s Esophagus: A Systematic Review and Meta-Analysis. Am J Epidemiol. 2008; 168: 237–49. 10.1093/aje/kwn121. . 18550563

[6] Ross-Innes CS, Becq J, Warren A, Cheetham RK, Northen H, O’Donovan M, Malhotra S, di Pietro M, Ivakhno S, He M, Weaver JMJ, Lynch AG, Kingsbury Z, et al. Whole-genome sequencing provides new insights into the clonal architecture of Barrett’s esophagus and esophageal adenocarcinoma. Nat Genet. 2015; 47: 1038-1046. 10.1038/ng.3357. . 26192915 PMC4556068

[7] Stachler MD, Taylor-Weiner A, Peng S, McKenna A, Agoston AT, Odze RD, Davison JM, Nason KS, Loda M, Leshchiner I, Stewart C, Stojanov P, Seepo S, et al. Paired exome analysis of Barrett’s esophagus and adenocarcinoma. Nat Genet. 2015; 47: 1047-1055. 10.1038/ng.3343. 26192918 PMC4552571

[8] Stachler MD, Camarda ND, Deitrick C, Kim A, Agoston AT, Odze RD, Hornick JL, Nag A, Thorner AR, Ducar M, Noffsinger A, Lash RH, Redston M, et al. Detection of Mutations in Barrett’s Esophagus Before Progression to High-Grade Dysplasia or Adenocarcinoma. Gastroenterology. 2018; 155: 156–67. 10.1053/J.GASTRO.2018.03.047. . 29608884 PMC6035092

[9] Rao DA, Berthier CC, Arazi A, Davidson A, Liu Y, Browne EP, Eisenhaure TM, Chicoine A, Lieb DJ, Smilek DE, Tosta P, Lederer JA, Brenner MB, et al A protocol for single-cell transcriptomics from cryopreserved renal tissue and urine for the Accelerating Medicine Partnership (AMP) RA/SLE network. bioRxiv. 2018; 275859 10.1101/275859.

[10] Gedye CA, Hussain A, Paterson J, Smrke A, Saini H, Sirskyj D, Pereira K, Lobo N, Stewart J, Go C, Ho J, Medrano M, Hyatt E, et al. Cell Surface Profiling Using High-Throughput Flow Cytometry: A Platform for Biomarker Discovery and Analysis of Cellular Heterogeneity. PLoS One. 2014; 9: e105602. 10.1371/journal.pone.0105602. . 25170899 PMC4149490

[11] Ecker BL, Taylor L, Zhang PJ, Furth EE, Ginsberg GG, McMillan MT, Datta J, Czerniecki BJ, Roses RE. HER3 Expression Is a Marker of Tumor Progression in Premalignant Lesions of the Gastroesophageal Junction. PLoS One. 2016; 11: e0161781. 10.1371/journal.pone.0161781. . 27559738 PMC4999185

[12] Brandtner AK, Quante M. Risk prediction in Barrett’s esophagus – aspects of a combination of molecular and epidemiologic biomarkers reflecting alterations of the microenvironment. Scand J Clin Lab Invest. 2016; 76: S63–9. 10.1080/00365513.2016.1210327. 27467504

[13] Fassan M, Mastracci L, Grillo F, Zagonel V, Bruno S, Battaglia G, Pitto F, Nitti D, Celiento T, Zaninotto G, Fiocca R, Rugge M. Early HER2 dysregulation in gastric and oesophageal carcinogenesis. Histopathology. 2012; 61: 769–76. 10.1111/j.1365-2559.2012.04272.x. . 22882541

[14] Quante M, Bhagat G, Abrams JA, Marache F, Good P, Lee MD, Lee Y, Friedman R, Asfaha S, Dubeykovskaya Z, Mahmood U, Figueiredo JL, Kitajewski J, et al. Bile Acid and Inflammation Activate Gastric Cardia Stem Cells in a Mouse Model of Barrett-Like Metaplasia. Cancer Cell. 2012; 21: 36–51. 10.1016/j.ccr.2011.12.004. . 22264787 PMC3266546

[15] Fitzgerald RC, Onwuegbusi BA, Bajaj-Elliott M, Saeed IT, Burnham WR, Farthing MJ. Diversity in the oesophageal phenotypic response to gastro-oesophageal reflux: immunological determinants. Gut. 2002; 50: 451-9. 10.1136/gut.50.4.451. . 11889061 PMC1773186

[16] Moons LM, Kusters JG, Bultman E, Kuipers EJ, van Dekken H, Tra WM, Kleinjan A, Kwekkeboom J, van Vliet AH, Siersema PD. Barrett’s oesophagus is characterized by a predominantly humoral inflammatory response. J Pathol. 2005; 207: 269–76. 10.1002/path.1847. . 16177953

[17] Martínez-Lostao L, Anel A, Pardo J. How Do Cytotoxic Lymphocytes Kill Cancer Cells? Clin Cancer Res. 2015; 21:5047-56. 10.1158/1078-0432.CCR-15-0685 . 26567364

[18] Van Pel A, van der Bruggen P, Coulie PG, Brichard VG, Lethé B, van den Eynde B, Uyttenhove C, Renauld JC, Boon T. Genes coding for tumor antigens recognized by cytolytic T lymphocytes. Immunol Rev. 1995; 145: 229–50. 10.1111/j.1600-065X.1995.tb00084.x. . 7590828

[19] Fouad YA, Aanei C. Revisiting the hallmarks of cancer. Am J Cancer Res. 2017; 7: 1016–36. . 28560055 PMC5446472

[20] Munn LL. Cancer and inflammation. Wiley Interdiscip Rev Syst Biol Med. 2017; 9: e1370. 10.1002/wsbm.1370. . 27943646 PMC5561333

[21] O’Byrne KJ, Dalgleish AG. Chronic immune activation and inflammation as the cause of malignancy. Br J Cancer. 2001; 85: 473–83. 10.1054/bjoc.2001.1943. . 11506482 PMC2364095

[22] Derks S, Nason KS, Liao X, Stachler MD, Liu KX, Liu JB, Sicinska E, Goldberg MS, Freeman GJ, Rodig SJ, Davison JM, Bass AJ. Epithelial PD-L2 Expression Marks Barrett’s Esophagus and Esophageal Adenocarcinoma. Cancer Immunol Res. 2015; 3: 1123–9. 10.1158/2326-6066.CIR-15-0046. . 26081225 PMC4596773

[23] Almhanna K, Rosa M, Henderson-Jackson E, Jiang K, Shamekh R, Sayegh Z, Malafa MP, Coppola D. Her-2 Expression in Gastroesophageal Intestinal Metaplasia, Dysplasia, and Adenocarcinoma. Appl Immunohistochem Mol Morphol. 2016; 24: 633–8. 10.1097/PAI.0000000000000243. . 26186253 PMC7771552

[24] Rossi E, Grisanti S, Villanacci V, Casa D Della, Cengia P, Missale G, Minelli L, Buglione M, Cestari R, Bassotti G. HER-2 overexpression/amplification in Barrett’s oesophagus predicts early transition from dysplasia to adenocarcinoma: a clinico-pathologic study. J Cell Mol Med. 2009; 13: 3826–33. 10.1111/j.1582-4934.2008.00517.x. . 19292734 PMC4516530

[25] McDivitt RW, Stone KR, Meyer JS. A method for dissociation of viable human breast cancer cells that produces flow cytometric kinetic information similar to that obtained by thymidine labeling. Cancer Res. 1984; 44: 2628–33. . 6327021

[26] O’Hare MJ, Ormerod MG, Monaghan P, Lane EB, Gusterson BA. Characterization in vitro of luminal and myoepithelial cells isolated from the human mammary gland by cell sorting. Differentiation. 1991; 46: 209–21. 10.1111/j.1432-0436.1991.tb00883.x. . 1833254

[27] Péchoux C, Gudjonsson T, Rønnov-Jessen L, Bissell MJ, Petersen OW. Human Mammary Luminal Epithelial Cells Contain Progenitors to Myoepithelial Cells. Dev Biol. 1999; 206: 88–99. 10.1006/dbio.1998.9133. . 9918697

[28] Snowden E, Porter W, Hahn F, Ferguson M, Tong F, Parker JS, Middlebrook A, Ghanekar S, Dillmore WS, Blaesius R. Immunophenotyping and Transcriptomic Outcomes in PDX-Derived TNBC Tissue. Mol Cancer Res. 2017; 15: 429–38. 10.1158/1541-7786.MCR-16-0286-T. . 28039356

[29] Ram M, Najafi A, Shakeri MT. Classification and Biomarker Genes Selection for Cancer Gene Expression Data Using Random Forest. Iran J Pathol. 2017; 12: 339–47. https://ijp.iranpath.org/article_27990.html. 29563929 PMC5844678

