# Correction: Flow based single cell analysis of the immune landscape distinguishes Barrett’s esophagus from adjacent normal tissue

**DOI:** 10.18632/oncotarget.27161

**Published:** 2019-08-20

**Authors:** Moen Sen, Friedrich Hahn, Taylor A. Black, Maureen DeMarshall, Warren Porter, Eileen Snowden, Stephanie S. Yee, Frances Tong, Mitchell Ferguson, Emylee N. Fleshman, Hiroshi Nakagawa, Gary W. Falk, Gregory G. Ginsberg, Michael L. Kochman, Rainer Blaesius, Anil K. Rustgi, Erica L. Carpenter

**Affiliations:** ^1^ Division of Hematology and Oncology, Department of Medicine, Abramson Cancer Center, Perelman School of Medicine, University of Pennsylvania, Philadelphia, Pennsylvania, USA; ^2^ Department of Genomic Sciences, BD Technologies and Innovation, Research Triangle Park, Durham, North Carolina, USA; ^3^ Division of Gastroenterology, Department of Medicine, Abramson Cancer Center, Perelman School of Medicine at the University of Pennsylvania, Philadelphia, Pennsylvania, USA; ^*^ These co-first authors have contributed equally to this work; ^**^ Co-corresponding authors

**This article has been corrected:** Authors Anil K. Rustgi and Erica L. Carpenter contributed equally as corresponding authors. The footnote to the author list indicating both individuals as co-corresponding authors was unintentionally omitted. The proper footnote has now been added as shown below:

^**^ Co-corresponding authors

Original article: Oncotarget. 2019; 10:3592–3604. 3592-3604 . https://doi.org/10.18632/oncotarget.26911

