# Supplementary material for: Flow based single cell analysis of the immune landscape distinguishes Barrett’s esophagus from adjacent normal tissue
Source: Oncotarget. 2019 Jun 4;10(38):3592–604. doi: 10.18632/oncotarget.26911 (PMC6557213; doi:10.18632/oncotarget.26911)
Supplement: Supplementary file 1 [file oncotarget-10-3592-s001.pdf]

Flow based single cell analysis of the immune landscape distinguishes Barrett's esophagus from adjacent normal tissue

SUPPMENTARY MATERIALS

Supplementary Table 1: Individual patient characteristics

Patient ID	Age	Sex	Prague score	Pathology
UPENN31	79	F	C6M7	Barrett's esophagus - Low Grade Dysplasia
UPENN32	70	M	C4M5	Barrett's esophagus - Intestinal Metaplasia
UPENN35	56	M	C9M10	Barrett's esophagus - Intestinal Metaplasia
UPENN36	54	F	C3M4	Barrett's esophagus - Intestinal Metaplasia
UPENN37	81	M	C1M2	Barrett's esophagus - Intestinal Metaplasia
UPENN38	64	M	C4M7	Barrett's esophagus - High Grade Dysplasia
UPENN41	60	M	C9M10	Barrett's esophagus - Intestinal Metaplasia
UPENN42	62	M	C0M1	Barrett's esophagus - Intestinal Metaplasia
UPENN44	54	F	C2M4	Barrett's esophagus - Intestinal Metaplasia
UPENN45	55	M	C0M8	Barrett's esophagus - High Grade Dysplasia with adenocarcinoma
UPENN46	72	M	C4M7	Barrett's esophagus - Intestinal Metaplasia
UPENN47	53	F	C1M2	Barrett's esophagus - High Grade Dysplasia with adenocarcinoma

Supplementary Table 2: Antibodies used for immunophenotyping BE and normal adjacent squamous tissue

mAB	mAB clone	Fluorochrome	Catalog number	Company
EpCAM	EBA-1	PerCP-Cy5.5	347199	BD
CD24	ML5	PE-CF594	5624056	BD
CD44	G44-26	Alexa Fluor 700	561289	BD
CD49f	GoH3	BV421	562582	BD
HER2	Neu 24.7	PE	340552	BD
CD133	W6B3C1	APC	566596	BD
CD90	5E10	PE-Cy7	561558	BD
CD166	3A6	PE/BV786	559263/564939	BD
CD29	MAR4	APC/BV605	559883/743784	BD
CD45	HI30	APC-H7	560178	BD
CD3	UCHT1	FITC	561807	BD
CD4	RPA-T4	PE-CF594	562281	BD
CD8	RPA-T8	Alexa Fluor 700	557945	BD
CD127	HIL-7R-M21	Alexa Fluor 647	558598	BD
CD56	NCAM 16.2	PE	340363	BD
CD25	2A3	PE-Cy7	335769	BD
CD19	SJ25C1	PerCP-Cy5.5	340951	BD
CD14	M5E2	FITC	555397	BD
CD184	12G5	APC/BV711	555976/740799	BD
Live Dead	n/a	Aqua	L10119	Invitrogen
Hoechst 33342	n/a	UV450	H3570	Invitrogen

Supplementary Table 3: Accuracy as calculated by RFE for various sized marker panels

Number of Markers	Accuracy	Markers
5	0.8611	Live cells, EpCAM, CD45, CD3, CD133
6	0.8500	Live cells, EpCAM, CD45, CD3, CD133, CD29
7	0.8500	Live cells, EpCAM, CD45, CD3, CD133, CD29, B Cells
8	0.8611	Live cells, EpCAM, CD45, CD3, CD133, CD29, B Cells, CD49f
9	0.8611	Live cells, EpCAM, CD45, CD3, CD133, CD29, B Cells, CD49f, Her2
10	0.8500	Live cells, EpCAM, CD45, CD3, CD133, CD29, B Cells, CD49f, Her2, CD4
11	0.8500	Live cells, EpCAM, CD45, CD3, CD133, CD29, B Cells, CD49f, Her2, CD4, NK Cells
12	0.8167	Live cells, EpCAM, CD45, CD3, CD133, CD29, B Cells, CD49f, Her2, CD4, NK Cells, CD44
13	0.8778	Live cells, EpCAM, CD45, CD3, CD133, CD29, B Cells, CD49f, Her2, CD4, NK Cells, CD44, CD166
14	0.8556	Live cells, EpCAM, CD45, CD3, CD133, CD29, B Cells, CD49f, Her2, CD4, NK Cells, CD44, CD166, CD8
19	0.8444	Live cells, EpCAM, CD45, CD3, CD133, CD29, B Cells, CD49f, Her2, CD4, NK Cells, CD44, CD166, CD8, Treg, CD24, CD90, CD184, Monos
